# *Trichomonas vaginalis* Induces Production of Proinflammatory Cytokines in Mouse Macrophages Through Activation of MAPK and NF-κB Pathways Partially Mediated by TLR2

**DOI:** 10.3389/fmicb.2018.00712

**Published:** 2018-04-10

**Authors:** Ling Li, Xin Li, Pengtao Gong, Xichen Zhang, Zhengtao Yang, Ju Yang, Jianhua Li

**Affiliations:** Key Laboratory of Zoonosis, Ministry of Education, College of Veterinary Medicine, Jilin University, Changchun, China

**Keywords:** *Trichomonas vaginalis*, TLR2, TLR2^-/-^, MAPK, NF-κB, cytokines

## Abstract

Trichomoniasis, caused by *Trichomonas vaginalis* infection, is the most prevalent sexually transmitted disease in female and male globally. However, the mechanisms by innate immunity against *T. vaginalis* infection have not been fully elucidated. Toll-like receptor2 (TLR2) has been shown to be involved in pathogen recognition, innate immunity activation, and inflammatory response to the pathogens. Nonetheless, the function of TLR2 against *T. vaginalis* remains unclear. In the present study, we investigated the role of TLR2 in mouse macrophages against *T. vaginalis*. RT-qPCR analysis revealed that *T. vaginalis* stimulation increased the gene expression of TLR2 in wild-type (WT) mouse macrophages. *T. vaginalis* also induced the secretion of IL-6, TNF-α, and IFN-γ in WT mouse macrophages, and the expression of these cytokines significantly decreased in TLR^2-/-^ mouse macrophages and in WT mouse macrophages pretreated with MAPK inhibitors SB203580 (p38) and PD98059 (ERK). Western blot analysis demonstrated that *T. vaginalis* stimulation induced the activation of p38, ERK, and p65 NF-κB signal pathways in WT mouse macrophages, and the phosphorylation of p38, ERK, and p65 NF-κB significantly decreased in TLR2^-/-^ mouse macrophages. Taken together, our data suggested that *T. vaginalis* may regulates proinflammatory cytokines production by activation of p38, ERK, and NF-κB p65 signal pathways via TLR2 in mouse macrophages. TLR2 might be involved in the defense and elimination of *T. vaginalis* infection.

## Introduction

Trichomoniasis is caused by *Trichomonas vaginalis* infection. As the most prevalent sexually transmitted disease worldwide, about 280 million people are infected with *T. vaginalis* every year (World Health Organization, 2012). In addition to causing serious discomfort, trichomoniasis has also been linked to vaginitis, preterm delivery, low birth weight, infertility, and cervical cancer (Grodstein et al., 1993; Cotch et al., 1997; Viikki et al., 2000). In infected men, *T. vaginalis* can be parasitic in prostate, epididymis or foreskin capsule and cause male urinary tract disease (Seña et al., 2007; Johnston and Mabey, 2008; Ryan et al., 2011). Although at least 80% of *T. vaginalis* infections are asymptomatic, epidemiological studies have also found that trichomoniasis is a risk factor of human immunodeficiency virus transmission (Rottingen et al., 2001). It is obvious that *T. vaginalis* infection has important medical, social, and economical implications. However, the mechanisms by innate immunity against *T. vaginalis* infection have not been fully elucidated.

The innate immunity plays a crucial role on the elimination of pathogen infections and defense against invading microorganisms. TLRs are a well-known group of pattern recognition receptors that recognize conserved pathogen-associated molecular patterns. Different TLR family members are expressed by a variety of cells in many animal species which are critical in generating innate immune responses to multiple stimuli (Aderem and Ulevitch, 2000; Anderson, 2000; Akira et al., 2001). Inflammatory responses mediated by TLRs can be triggered by a variety of pathogens, including parasite, bacteria, fungi, and virus (Kawai and Akira, 2005; Oliveira-Nascimento et al., 2012). TLR activation not only leads to inflammatory responses but is also involved in the development of adaptive immunity for specific antigens. Stimulation of adaptive immunity can promote a series of host immune defense mechanisms, such as the activation of mitogen-activated protein kinases (MAPKs) and the secretion of proinflammatory cytokines which participate in the elimination of pathogens (Takeda et al., 2003). Activation of TLR in turn activates downstream MAPK signal pathways especially the extracellular signal-regulated kinase p38 and ERK which regulates a variety of cellular responses, such as inflammatory, differentiation, and apoptosis. Previous studies have demonstrated that NF-κB participates in the modulation of inflammatory responses during early infection stage, and parasites like *Leishmania* and *Toxoplasma gondii* could interfere with the activation of the NF-κB signal pathways (Shapira et al., 2005; Reinhard et al., 2012).

TLR2 recognizes PAMPs and informs immune cells of invading pathogens. TLR2 activation leads to the proinflammatory cytokine production through the activation of MAPK, AKT, and NF-κB signal pathways (Soilleux et al., 2002; Vasselon et al., 2002; Asehnoune et al., 2005). TLR2 can be activated by glycosylphosphatidylinositols (GPIs) presented on some protozoa and participates in the host defense against parasite infection (Oliveira-Nascimento et al., 2012), including *Toxoplasma gondii* and *Trypanosoma cruzi* (Campos et al., 2001; Debierre-Grockiego et al., 2007) The expression of TLR2 has been confirmed in various cells, such as endothelial cells, epithelial cells, and macrophages (Flo et al., 2001; Yadav and Schorey, 2006; Brzezińska-Błaszczyk and Wierzbicki, 2010). Macrophages are important for innate immune system during infection and are involved in a series of inflammatory reactions, such as the secretion of IL-6, TNF-α, and IFN-γ (Gessani and Belardelli, 1998; Butcher and Denkers, 2002; Goral et al., 2004). Previous studies have shown that *T vaginalis* infection in women caused high level production of proinflammatory cytokines including IL-6, IL-1β, and TNF-α in cervicovaginal mucosa (Riezzo et al., 2007; Han et al., 2009). However, the role of TLR2 in the host defense against *T. vaginalis* remains unclear.

In this study, we examined the expression of TLR2; the activation of p38, ERK, and NF-κB signal pathways; the secretion of IL-6, TNF-α, and IFN-γ in WT and TLR2^-/-^ mouse macrophages by RT-qPCR, western-blot, and ELISA, respectively.

## Materials and Methods

### *T. vaginalis* and Mouse Peritoneal Macrophages (PMϕ)

The *T. vaginalis* isolate used in this study was isolated from the vaginal secretions of trichomonas vaginitis in the gynecological clinic of the First Clinical Hospital of Jilin University (Zhao et al., 2007). *T. vaginalis* were cultivated in Diamond’s Typticase-yeast extract-maltose medium with 10% FBS at 37°C, only the late-logarithmic-phase trophozoites were used for the assays. Wild-type (WT) female C57BL/6 mice and TLR2^-/-^ mice were euthanized with over dose of ether (Rutkowski et al., 2007; Li et al., 2017) and soaked in 75% ethanol for 15 min, peritoneal cavity were flushed twice with 10 ml PBS (pH 7.4), then cell suspension were centrifuged at 1,000 *g* for 10 min, and washed twice with PBS.The primary culture cell viability of macrophages obtained by the lavage fluid was determined by trypan blue method (>99%). 3 × 10^6^ cells were plated in a well of 6-well tissue culture plates (JET BIOFIL, China) in 1 ml RPMI 1640 containing 10% FBS, 2 mM L-glutamine, 100 U/ml penicillin, and 100 mg/ml streptomycin and incubated overnight at 37°C with 5% CO_2_. Cells were washed twice with PBS to remove the non-adherent cells before stimulation.

### Real-Time PCR Measurement for TLR2 Gene Expression

3 × 10^6^ WT peritoneal macrophages (PMϕ) were co-incubated for 2 h with 1 × 10^6^ *T. vaginalis* or Pam3Cys-Ser-(Lys) 4 (Pam3CSK_4_) (TLR2/TLR1 Agonist, InvivoGen, United States, 10 μg/ml) (Summers et al., 2014) in 1 ml RPMI 1640 containing 10% FBS, 2 mM L-glutamine, 100 U/ml penicillin, and 100 mg/ml streptomycin at 37°C with 5% CO_2_. After treatments, cell culture supernatants were removed and the cells were washed twice with RPMI 1640. Total RNA was isolated from the cells using the TRIzol Reagent (Invitrogen, United States). First-strand cDNA was synthesized by reverse transcription using the total RNA Transcript II reverse transcriptase (TransGen Biotech Company, Beijing, China). RNA expression levels of the analyzed genes were normalized to the amount of β-actin. The PCR program ran for 30 cycles with three steps: 95°C for 3 min; 95°C for 45 s, 60°C for 45 s, and 72°C for 45 s; 72°C for 10 min. Melting curves were acquired after PCR to ensure the homogeneity of the PCR products. All primers were synthesized by Sangon (Shanghai, China), and their sequences were as follows:

β-actin, sense: 5′-TGCTGTCCCTGTATGCCTCT-3′, antisense: 5′-GGTCTTTACGGATGTCAACG-3′; TLR2, sense: 5′-CCCACTTCAGGCTCTTTGAC-3′, antisense: 5′-GCCACTCCAGGTAGGTCTTG-3′.

### Detection of Cytokines by ELISA

3 × 10^6^ WT or TLR2^-/-^ PMϕ were co-incubated with 1 × 10^6^ *T. vaginalis* or Pam3CSK4 (10 ug/ml) for 18 h in RPMI 1640 containing 2% FBS, 2 mM L-glutamine, 100 U/ml penicillin, and 100 mg/ml streptomycin at 37°C with 5% CO_2_. In the inhibition experiment, WT PMϕ were pretreated with specific inhibitor of p38 (SB203580; 30 μM), ERK (PD98059; 40 μM) (Sigma-Aldrich, United States) for 1 h at 37°C with 5% CO_2_ and the viability of the macrophages was determined by trypan blue method (>95%). Then the PMϕ were co-incubated with *T. vaginalis* in cell culture medium containing 2% FBS. Culture supernatants were collected and IL-6, TNF-α, and IFN-γ levels were analyzed by ELISA Ready-SET-Go kits (eBioscience, San Diego, CA, United States) according to the manufacturer.

### Western Blot Analysis

3 × 10^6^ WT or TLR2^-/-^ PMϕ in monolayer were co-incubated with 1 × 10^6^ *T. vaginalis* for 0.5, 1, 2, and 4 h. In parallel experiments, WT PMϕ were pretreated with specific inhibitor of p38 (SB203580; 30 μM), ERK (PD98059; 40 μM). Pam3CSK4 (10 ug/ml) were used to stimulate macrophages for the positive control (TLR2, NF-κB p65). Non-treated PMϕs were used as negative control. After the incubation, cells were harvested with 25 cm cell scrapers (SRASTEDT, Germany) and centrifuged at 12,000 *g* for 30 min at 4°C. The pellets were washed twice with sterile PBS, then treated with RIPA lysis buffer (1% NP-40, 0.5% Sodium deoxycholate, 0.1% SDS) (BOSTER, China) containing proteinase inhibitor (1/100) and phosphatase inhibitor (1/100) (Sangon Biotech, Shanghai, China). Protein concentrations were measured using the Bradford protein-quantification assay. 30 μg of sample protein/lane was separated with 10% SDS-PAGE electrophoresis, then transferred to polyvinyldifluoride membranes (Millipore, Bedford, MA, United States) and blocked in 5% skim milk in TBST for 2 h at room temperature. The membranes were incubated overnight at 4°C with primary rabbit antibodies anti-TLR2 (1/1,000), anti-ERK (1/1,000), anti-p38 (1/1,000), anti-IκBα (1/1,000), anti-p65 (1/1,000), anti-phospho-ERK (1/1,000), anti-phospho-p38 (1/1,000), anti-phospho-IκBα (1/1,000) and anti-phospho-p65 (1/1,000) (Cell Signaling Technology, United States) and mouse anti-β-actin (1/2,000) (Bioss, United States). After 1 h of washing with TBST, membranes were incubated with secondary HRP conjugated goat anti-rabbit IgG or anti-mouse IgG (Cell Signaling Technology, dilution 1/5,000) for 1 h at room temperature and washed three times with TBST. Bands were detected using enhanced chemiluminescence (Vigorous, Beijing, China). The Relative Gray Value (target protein/internal reference) of the western blot results were analyzed by Image J.

### Subcellular Localization of NF-κB p65

The localization of NF-κB p65 were determined after 7.5 × 10^5^ PMϕ cultivated on glass coverslips in a well of 24-well culture plates co-incubated with 1 × 10^6^ *T. vaginalis* or Pam3CSK4 (10 ug/ml) at 37°C. After stimulation for 0 or 1 h, cells were washed twice with PBS, then fixed with 4% paraformaldehyde (diluted in PBS) for 15 min at room temperature and washed three times with PBS. The macrophages were permeabilized with 0.25% Triton X-100 for 10 min, then incubated with mouse anti-phosphor NF-κB p65 (Santa Cruz, CA, United States) at a 1:100 dilution at 4°C overnight. Cells were then washed and incubated with FITC-conjugated goat anti-mouse IgG (Proteintech, United States, dilution 1/5,000) secondary antibody for 1 h at room temperature. The cells were washed, and the coverslips were stained with DAPI at room temperature for 5 min. NF-κB p65 localization was observed using a Zeiss LSM 710 confocal microscope equipped with a 63X, 1.4-NA, oil-immersion objective (Carl Zeiss).

### Statistical Analysis

All data were presented as mean ± SD of triplicate experiments. GraphPad Prism 5 (GraphPad Software, Inc., San Diego, CA, United States) was utilized to analyze the data detected by ELISA. Image J was used to analyze the results of western blot and confocal microscope. SPSS version 19.0 (SPSS, Inc., Chicago, IL, United States) was used for the statistical analysis using ANOVA followed by Tukey test. *p*-Values < 0.05 were considered statistically significant.

## Results

### TLR2 Activation and Proinflammatory Cytokine Production in PMϕ Induced by *T. vaginalis*

**FIGURE 1:**
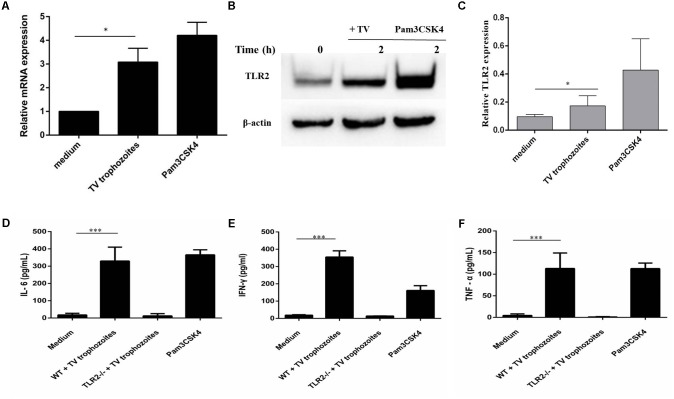
*Trichomonas vaginalis* induces cytokines secretion in a TLR2-dependent way in mouse peritoneal macrophages. RT-qPCR analysis for TLR2 in total RNA isolated from mouse peritoneal macrophages incubated in medium alone, with *T. vaginalis* or Pam3CSK4 (10 μg/ml), respectively. RT-qPCR demonstrated that *T. vaginalis* stimulation significantly enhances TLR2 gene expression in WT macrophages **(A)**. WT macrophages were co-incubated with *T. vaginalis* or Pam3CSK4 for 2 h **(B)**. Relative Gray analysis of western blot **(C)**. WT and TLR2^-/-^ mouse peritoneal macrophages were co-incubated with *T. vaginalis*. The levels of IL-6, TNF-α, and IFN-γ in cell culture supernatant were detected by ELISA. Compared with TLR2^-/-^ mouse peritoneal macrophages, the production of IL-6, TNF-α, and IFN-γ in WT macrophages were significantly increased **(D–F)**. Data are expressed as the mean ± SD from three separate experiments (^∗^*p* < 0.05, ^∗∗^*p* < 0.01, ^∗∗∗^*p* < 0.001).

RT-qPCR and western blot results showed that TLR2 gene expression increased in WT PMϕ co-incubated with *T. vaginalis* for 2 h compared with PMϕ without *T. vaginalis* (**Figures 1A,B**). The most significant TLR2 expression was detected in Pam3CSK4 (10 ug/ml) stimulated macrophages. Relative Gray analysis further confirmed this result (**Figure 1C**). Secretion of IL-6 (**Figure 1D**), IFN-γ (**Figure 1E**), and TNF-α (**Figure 1F**) increased in WT PMϕ with *T. vaginalis* co-incubation compared to TLR2^-/-^ PMϕ with *T. vaginalis* or WT PMϕ without *T. vaginalis*.

### *T. vaginalis* Activated p38 and ERK Signal Pathways in WT PMϕ

**FIGURE 2:**
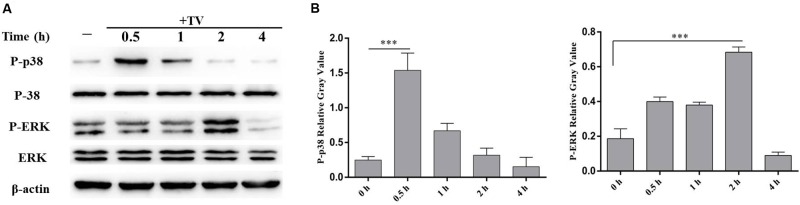
*Trichomonas vaginalis* activates p38 and ERK signal pathways in WT mouse peritoneal macrophages. WT macrophages were co-incubated with *T. vaginalis* for different times (0, 0.5, 1, 2, and 4 h), phosphorylation of p38 and ERK were detected by western blot **(A)**. Relative Gray analysis of western blot **(B)**. The phosphorylated of p38 (at 0.5 h) and ERK (at 2 h) were observed obviously. Data are expressed as the mean ± SD from three separate experiments (^∗^*p* < 0.05, ^∗∗^*p* < 0.01, ^∗∗∗^*p* < 0.001).

The phosphorylation of p38 and ERK were detected by western blot after co-incubation of WT PMϕ with *T. vaginalis* for 0.5 and 2 h. The phosphorylation of p38 peaked at 0.5 h and returned to baseline at 2 h, while phosphorylated ERK peaked at 2 h in WT PMϕ with *T. vaginalis* stimulation compared to control PMϕ without *T. vaginalis* stimulation (**Figure 2**).

### *T. vaginalis* Induced Cytokines Secretion in PMϕ Through Phosphorylation of p38 and ERK via TLR2

**FIGURE 3:**
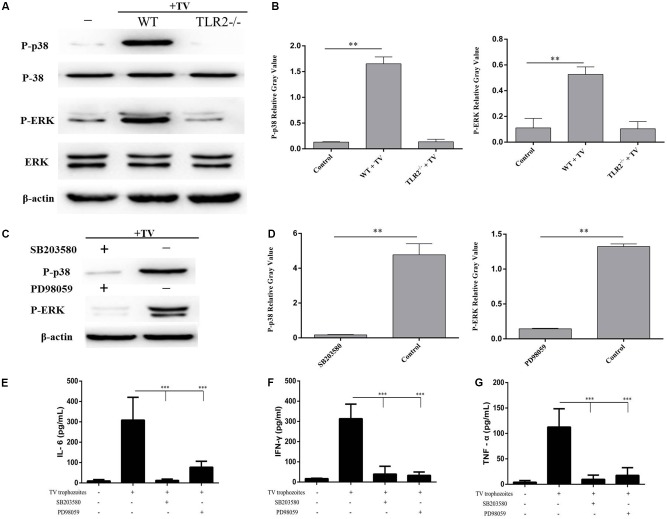
*Trichomonas vaginalis* induces cytokines production regulated by p38 and ERK via TLR2. Phosphorylationof p38 and ERK in TLR2^-/-^ mouse peritoneal macrophages were significantly reduced after co-incubated with *T. vaginalis* for 0.5 h (p38) or 2 h (ERK) compared to in WT mouse peritoneal macrophages **(A)**. Inhibitors of p38 (SB203580; 30 μM) or ERK (PD98059; 40 μM) were used to pretreated WT mouse peritoneal macrophages for 1 h before co-incubated by *T. vaginalis*. Phosphorylation of p38 and ERK in SB203580 and PD98059 pre-treated WT macrophages **(C)**. Relative Gray analysis of western blot **(B,D)**. The production of IL-6, TNF-α, and IFN-γ induced by *T. vaginalis* were significantly inhibited by the inhibitors compared to in WT mouse peritoneal macrophages **(E–G)**. Data are expressed as the mean ± SD from three separate experiments (^∗^*p* < 0.05, ^∗∗^*p* < 0.01, ^∗∗∗^*p* < 0.001).

To investigate whether the phosphorylation of p38 and ERK induced by *T. vaginalis* was mediated through TLR2, TLR2^-/-^ PMϕ were incubated with *T. vaginalis* for 0.5 or 2 h. The phosphorylation of p38 and ERK in TLR2^-/-^ PMϕ was significantly reduced in TLR2^-/-^ PMϕ with *T. vaginalis* co-incubation compared to WT PMϕ with *T. vaginalis* or WT PMϕ without *T. vaginalis*. These data demonstrated that *T. vaginalis* induced phosphorylation of p38 and ERK via TLR2 (**Figure 3A**).

To estimate the role of p38 and ERK signaling pathways in the regulation of IL-6, TNF-α, and IFN-γ productions, two MAPK inhibitors SB203580 (p38) and PD98059 (ERK) were used to pretreat WT PMϕ for 60 min at 37°C. Phosphorylation of p38 and ERK in inhibitor pretreated PMϕ (**Figure 3C**). Relative Gray analysis of western blot (**Figures 3B,D**). Macrophages with or without inhibitor treatments were incubated with *T. vaginalis* for 18 h, then cytokines in different samples were measured by ELISA. The results indicated that the production of IL-6, TNF-α, and IFN-γ induced by *T. vaginalis* were significantly reduced in WT PMϕ treated with the two inhibitors compared to PMϕ without inhibitors (**Figures 3E–G**). These results indicated that p38 and ERK signaling were activated via TLR2 which in turn upregulated the expression of IL-6, TNF-α, and IFN-γ in PMϕ in response to *T. vaginalis*.

### *T. vaginalis* Induced Translocation of NF-κB p65 to Nucleus via TLR2 in WT PMϕ

**FIGURE 4:**
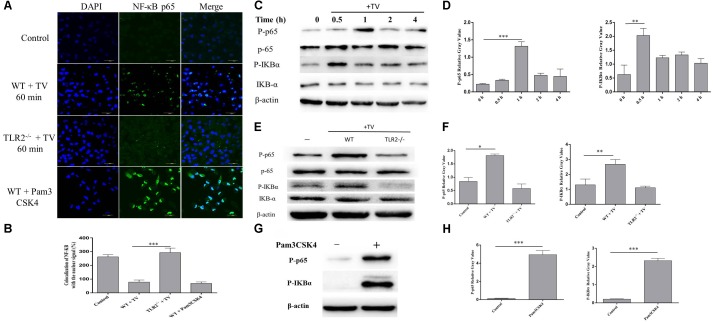
*Trichomonas vaginalis* induces changes of subcellular localization of NF-κB p65 via TLR2. Confocal microscopy analysis revealed the effects of *T. vaginalis* or Pam3CSK4 on the translocation of NF-κB p65 from the cytoplasm to the nucleus in WT macrophages after the incubation with *T. vaginalis* for 1 h **(A)**. The percentage of colocalization of NF-κB with the nuclear signal under the different treatments **(B)**. WT mouse macrophages were incubated with *T. vaginalis* for different times (0–4 h), cell lysates were used for western blot analysis **(C)**. WT and TLR2^-/-^ mouse macrophages were incubated with *T. vaginalis* for 1 h, phosphorylation of NF-κB p65 and IκBα was detected by western blot **(E)**. Phosphorylation of NF-κB p65 in Pam3CSK4 treated macrophages **(G)**. Relative Gray analysis of western blot **(D,F,H)**.

The activation of NF-κB by *T. vaginalis* was detected by immunofluorescence staining and western blot. Phosphorylated NF-κB p65 was observed in the WT PMϕ nucleus after co-incubation with *T. vaginalis* (**Figure 4A**) and its phosphorylation increased, as well as of IKB-α (**Figure 4C**). The percentage of colocalization of p65 with nuclei signal (**Figure 4B**). To investigate whether the activation of NF-κB p65 was via TLR2, TLR2^-/-^ PMϕ was co-incubated with *T. vaginalis* and NF-κB p65 localization was observed. NF-κB p65 was located primarily in the cytoplasm of TLR2^-/-^ PMϕ after co-incubation with *T. vaginalis* (**Figure 4A**). In addition, western blot analysis also showed that phosphorylated NF-κB p65 and IκB-α in TLR2^-/-^ PMϕ with *T. vaginalis* were significantly reduced compared to WT PMϕ (**Figure 4E**). Pam3CSK4 (10 ug/ml) stimulated macrophages showed the most significant translocation and phosphorylation of NF-κB p65 (**Figure 4G**). Relative Gray analysis of western blot (**Figures 4D,F,H**). These data demonstrated that *T. vaginalis* may induce the phosphorylation of NF-κB p65 via TLR2 in WT PMϕ.

## Discussion

Toll-like receptors are important molecules for the recognition of different pathogens, including parasites, bacteria, viruses, and fungi. Components of microorganisms were recognized by different TLRs, which result in the activation of intracellular signals and cytokine production by leukocytes and other cells that are necessary for pathogenic microorganisms elimination (Medzhitov, 2001; Kawai and Akira, 2007). TLR2 mediates cell activation by the recognition of microorganisms and microbial products, and also is involved in the defense and elimination of parasites infection, including *Plasmodium falciparum* (Zhu et al., 2011), *Toxoplasma gondii* (Mun et al., 2003), and *Trypanosoma cruzi* (Campos et al., 2001). HeLa cells treated with *T. vaginalis* showed significant up-regulation of TLR2, TLR4, and TLR9 (Chang et al., 2006). In this study, we analyzed TLR2, TLR4, and TLR9 gene expression in WT macrophages and the results from RT-qPCR and western blot demonstrated that *T. vaginalis* stimulation significantly enhanced TLR2 gene expression in WT macrophages.

Previous studies also implicated that *T. vaginalis* infection induced inflammatory responses in epithelial (Yang et al., 2015), neutrophils (Ryu et al., 2004), and macrophages (Han et al., 2009). Proinflammatory cytokines such as IL-6 from human prostate epithelial cells and TNF-α from rat peritoneal mast cells have been demonstrated to be necessary for the *T. vaginalis* control and the initiation of subsequent adaptive immune response in the process of *T. vaginalis* infection (Im et al., 2011; Han et al., 2016). Furthermore, human monocyte-derived macrophages (HMDMs) co-cultured with *T. vaginalis* increased the production of proinflammatory cytokines, such as TNF-α, IL-1β, and IL-6 (Han et al., 2009). In our study, we found that the production of proinflammatory cytokines IL-6, TNF-α, and IFN-γ in WT macrophages were significantly higher than that in TLR2^-/-^ macrophages, which indicated that *T. vaginalis* induced proinflammatory cytokines production in macrophages was partially dependent on TLR2.

Innate immune system recognizes microorganism and activates a variety of signal transduction pathways which initiate the subsequent immune response (Beutler, 2009). The GPI from parasite could lead the activation of MAPK and NF-κB signal pathways via TLR2 (Gowda, 2007). Human macrophages and SiHa Cervical Mousosal Epithelial cells infected with *T. vaginalis* increased the activity of phospho NF-κB p65 and p38, ERK, and JNK, respectively (Han et al., 2009; Yang et al., 2015). HeLa cells treated with *T. vaginalis* showed significant up-regulation of TLR2, TLR4, TLR9, which was a p38 MAPK signaling pathway dependent process (Medzhitov, 2001). Our results showed that the phosphorylation of p38, ERK in WT macrophages significantly increased compared to in TLR2^-/-^ macrophages. Furthermore pretreatment with inhibitors of p38 and ERK obviously inhibited the production of IL-6, TNF-α, and IFN-γ. Taken together, our data suggested that *T. vaginalis* induced proinflammatory cytokines production in macrophages through the activation of MAPK via TLR2.

Nuclear transcription factor NF-κB, formed by five different Rel family proteins, is known to be crucial in the regulation of inflammatory gene transcription for both innate and adaptive immunity responses (Caamaño et al., 1999). Recent evidence demonstrated that a shift in NF-κB subunits from p50 to p65 heterodimer to p50 homodimers is associated with inflammation (Lawrence et al., 2001; Ashall et al., 2009), in addition, degradation of IkBα is a classical way for NF-κB p65 phosphorylation and translocation to the nucleus (Hayden and Ghosh, 2008). Studies on ultraviolet light (UV) and recombinant MPT83 derived from *Mycobacterium tuberculosis* showed that the activation of TLR2 was involved in the phosphorylation of NF-κB (Wang et al., 2011; Chen et al., 2012). The NF-κB activation also participated in the regulation of NO production and some NF-κB dependent proinflammatory gene expression can be up-regulated by CD40 (Song et al., 2011; Adesso et al., 2013). Western blot and immune fluorescence assays from the present study indicated that *T. vaginalis* induced the phosphorylation and accumulation in nucleus of NF-κB p65 in WT macrophages but not in TLR2^-/-^ macrophage. The results demonstrated that *T. vaginalis* could activate NF-κB signal pathway via TLR2 in mouse macrophage.

In summary, *T. vaginalis* could regulate pro-inflammation cytokines production by activation of p38, ERK, and NF-κB p65 signal pathways via TLR2 in mouse peritoneal macrophages. These data could enrichour understanding on host defense against *T. vaginalis* infection through innate immune system.

## Ethics Statement

WT C57BL/6 mice (Huafukang Experimental Animal Center, Beijing, China) and TLR2^-/-^ mice (Model Animal Research Center of Nanjing University, Nanjing, China) were housed in filter-top cages in an air-conditioned animal facility in the National Experimental Teaching Demonstration Centre of Jilin University (Changchun, China). Water and normal mouse food were given *ad libitum*. All animal experimental procedures were performed in strict accordance with the Regulations for the Administration of Affairs Concerning Experimental Animals approved through the State Council of People’s Republic of China (1988.11.1) and with approval of the Animal Welfare and Research Ethics Committee at Jilin University.

## Author Contributions

LL and PG drafted the main manuscript and performed the data analysis. LL and XL planned and performed the experiments. LL, ZY, JY, and JL were responsible for experimental design. XZ and JL responsible for guiding and supporting the experiments and manuscript revisions.

## Conflict of Interest Statement

The authors declare that the research was conducted in the absence of any commercial or financial relationships that could be construed as a potential conflict of interest.
